# Copper(II)/polyimide linked covalent organic framework as a powerful catalyst for the solvent‐free microwave irradiation‐based synthesis of 2,4,5‐trisubstituted imidazoles

**DOI:** 10.1002/ansa.202300012

**Published:** 2023-04-17

**Authors:** Mahnaz Sedaghat, Farid Moeinpour, Fatemeh S. Mohseni‐Shahri

**Affiliations:** ^1^ Department of Chemistry Bandar Abbas Branch Islamic Azad University Bandar Abbas Iran

**Keywords:** 2,4,5‐trisubstituted imidazoles, covalent organic framework, microwave, solvent‐free

## Abstract

Copper(II)/polyimide‐linked covalent organic frameworks under solvent‐free and microwave‐assisted conditions have been used in an efficient one‐pot protocol for the preparation of 2,4,5‐trisubstituted imidazoles via benzil, aromatic aldehydes and ammonium acetate. By applying solvent‐free conditions and microwave irradiation, three‐component condensation provides safe operations, low pollution, quick access to products, and an easy set‐up. As a result of its reusability, the catalyst can also be reutilized for many runs without missing any activity.

**Abbreviations**
COF: Covalent organic framework
SSA: Specific surface area
PI‐COFs: Covalent organic polyimide frameworks
PL‐COFs: Polyimide‐linked COFs
MEL: Melamine
BTCD: benzene‐1,2:4,5‐tetracarboxylic dianhydride

## 1 INTRODUCTION

Chemistry is art and chemical reactions are a world of art, where heat, light, catalyst, electricity, pressure, and sound can be used to perform chemical reactions. Therefore, according to the growing human need for energy and raw materials, the scope of chemical products has expanded, and they have found a special place in human daily life. A green chemistry approach is aimed at protecting environmental safety, health and economic efficiency while utilizing scientific and basic methods. As a general goal of this program, it asks for making more efficient and effective use of catalysts, replacing stoichiometric reactions with catalytic ones, and delivering new procedures of synthetic chemistry that do not depend upon raw materials and toxic solvents in order to be successful.^1^ In contrast, microwaves have been used to prepare a wide variety of organic and inorganic compounds, organic‐metallic complexes, polymers, and other chemical science subjects due to conducive reaction conditions in respect of high speed, product selectivity and increased reaction efficiency. Catalysts, which a large part of the world's industries are based on, facilitate the production of many chemical products and are considered the heart of many organic reactions. Nowadays, nanoparticles have attracted the attention of chemists as heterogeneous catalysts. Because nanocatalysts with their large surface area can carry active species.^2^, ^3^, ^4^, ^5^, ^6^, ^7^, ^8^

A covalent organic framework (COF) is a type of substance that creates two‐ or three‐dimensional construction by the reactions between organic precursors leading to covalent bonds to produce porous organic materials.^9^ Porous polymers are a group of polymers that have been the focus of many scholars due to the common characteristics of porous materials and polymers. These polymers can be synthesized by various methods for specific industrial applications, with high specific surface area (SSA) and designed pore sizes.^10^ Some significant constructional properties of porous polymers are the geometry, size and surface area of ​​the pores, and the structure of the polymer platform, including their composition, topology, and functionality. The organic structural units of COFs are/could be formed with such lightweight elements as C, B, O etc.^11^ These polymers create two‐dimensional and three‐dimensional structures with pore sizes of about 7–27 Å. Since the metal‐organic frameworks have a metallic structure with organic binders, they do not have good stability in acidic environments, and they are leached. This process is not observed in other porous organic polymers, because all their atoms are placed together with strong covalent bonds.^12^, ^13^ In these structures, there are covalent bonds between light atoms such as carbon‐carbon, carbon‐nitrogen, carbon‐oxygen, and boron‐oxygen. Properties such as stability heat, low density and adjustable pore size make COFs perform very well among porous organic structures in various applications. Among these applications, gas storage and separation, catalysis, adsorption, enrichment and purification of small molecules, molecular recognition, targeted drug delivery, conductive membranes and energy storage could be mentioned. Typically, these structures have a thermal stability of more than 300°C and are also compatible with many solvents. This makes them more stable than metal‐organic frameworks.^14^

The application of COFs as a heterogeneous ligand for immobilizing transition metal ions to support a variety of organic reactions has been shown to be promising.^15^, ^16^, ^17^, ^18^

Lately, covalent organic polyimide frameworks (PI‐COFs) with high thermal resistance, extraordinary mechanical quality, big pore sizes and predominant chemical stability have been created and prepared by incorporating linear and tetrahedral building blocks through the imidization response. While many studies have demonstrated the potential of PI‐COFs to be excellent functional materials for drug delivery,^19^ chemos and biosensors^20^ and organic dye depollution,^21^ the study of PI‐COFs as transition metal carriers has received relatively little attention.

Most biologically important molecules are rich in imidazole rings. Biochemical processes and pharmacological properties of compounds containing imidazole moieties are important. A type of imidazole that is used extensively in photography is 2,4,5‐triarylimidazole, which is also used in pharmaceutical formulations and as a fungicide and herbicide in certain applications as well as a growth regulator for plants.^22^ 2,4,5‐triarylimidazoles have been synthesized using a variety of methods that have been developed over the years. An aldehyde or an ammonium salt as the nitrogen source (most often ammonium acetate) in the presence of an ionic liquid,^23^ Yb(OTf)_3_,^24^ NiCl_2_.6H_2_O supported on acidic alumina,^25^ L‐cysteine,^26^ N‐bromosuccinimide,^27^ nano MgAl_2_O_4_,^28^ and CoFe_2_O_4_ ^29^ is used in the reported procedures for cyclocondensing 1,2‐diketones (benzoin or benzil).

Many methods of synthesizing 2,4,5‐triarylimidazoles have one or more restrictions, such as prolonged reaction times, the incidence of side reactions, harsh reaction conditions, low yields, use of corruptive chemicals and hazardous reagents, high‐boiling‐point solvents and cumbersome processing.^30^ Hence, an improved catalyst for the synthesis of 2,4,5‐triarylimidazoles is required to attain gentle reaction conditions, simple operation, economy, and selectivity.

**SCHEME 1:**
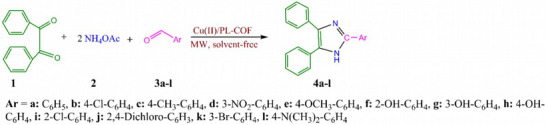
Cu(II)/polyimide‐linked covalent‐organic framework (PL‐COF) catalyst‐mediated synthesis of 2,4,5‐triarylimidazoles.

As part of our investigation plan in the context of nano‐catalysts,^31^, ^32^, ^33^, ^34^, ^35^, ^36^, ^37^ we report herein polyimide‐linked COFs (PL‐COFs) used as nano‐catalysts for the preparation of benzodiazepine derivatives. The generic procedure is given in Scheme 1.

## 2 EXPERIMENTAL SECTION

### 2.1 Materials

No further purification was performed on the analytical‐grade reagents, as they were obtained from Sigma.

### 2.2 Synthesis of PL‐COF

An equimolar ratio (20 mmol) of melamine (MEL, 2.522 g) and benzene‐1,2:4,5‐tetracarboxylic dianhydride (BTCD, 4.362 g) was milled in an opal mortar for 30 min, and finally transferred the resulting mixture to an alumina pot and annealed to 325°C for 4 h at a heating speed of 5°C/min. After air cooling to ambient temperature, the obtained product was rinsed at 50°C with water to eliminate the remaining MEL precursor and eventually desiccated at 80°C to afford PL‐COF.^38^

### 2.3 Synthetic method for Cu(II)/PL‐COF

100 mg of the synthesized PL‐COF was mixed with 30 mg Cu(CH_3_COO)_2_ dissolved in 30 ml absolute ethanol. During the four‐hour stirring period, the solution was kept at ambient temperature and then rinsed with absolute ethanol. Therefore, the resulting Cu(II)/PL‐COF was then activated by vacuum at 80°C.

### 2.4 2,4,5‐triarylimidazoles generic preparation method

In a Pyrex test tube, a mixture of benzil **1** (1 mmol, 0.210 g), ammonium acetate **2** (2 mmol, 0.154 g), aromatic aldehydes **3a‐l** (1 mmol) and 0.05 g catalyst was subjected to microwave radiation with a power of 180 W (MicroSynth) under solvent‐free conditions for convenient reaction time (2 min). It was monitored using TLC to determine how the reaction was progressing (normal hexane: ethyl acetate, 7:3). After termination of the reaction, the temperature of the reaction mixture was lowered to ambient temperature and 15 ml of hot ethanol was added. Then, to prepare for the next run, the Cu(II)/PL‐COF was removed from the cooled mixture by filtration, washed with a solution of acetone and then dried overnight. It was then transferred into a beaker the catalyst‐free reaction mixture that had been prepared. The colloidal solution obtained from the separation was crystallized with hot ethanol to obtain the pure product. The 2,4,5‐triarylimidazoles products were completely purified by recrystallization and a yellow precipitate was successfully obtained. It has been established that the structures of the products can be assigned via ^1^H‐NMR, and their melting points have been compared with those found earlier in the literature.

### 2.5 Selected spectral data

#### 2.5.1 2,4,5‐triphenyl‐1H‐imidazole (**4a**)

^1^HNMR (400 MHz, DMSO, ppm): δ: 12.71 (s, 1H, NH), 8.85‐6.92 (m, 15H).

#### 2.5.2 4,5‐diphenyl‐2‐(*p*‐tolyl)‐1H‐imidazole (**4c**)

^1^HNMR (400 MHz, DMSO, ppm): δ: 12.62 (s, 1H, NH), 7.94‐7.97 (d, 2H), 7.52‐7.21 (m, 14H,), 2.32 (s, 3H, CH_3_).

#### 2.5.3 4‐(4,5‐diphenyl‐1*H*‐imidazol‐2‐yl)phenol (**4h**)

^1^HNMR (400 MHz, DMSO, ppm): δ: 12.42 (s, 1H, NH), 9.72 (s, 1H, OH), 7.87‐7.85 (d, 2H), 7.51‐7.22 (m, 13H), 6.82‐6.80 (d, 2H).

### 2.6 Characterizations

Shimadzu spectrometers (8400s, Kyoto, Japan) were used to register Fourier‐transform infrared (FT‐IR) spectra with KBr pellets in the 400–4000 cm^−1^ range. X‐ray diffractometers (Philips) were used to study the phase crystallinity of the catalyst using Cu Kα radiation (λ = 1.54 Å). The morphology of nanopowders was characterized by using field‐emission scanning electron microscopy (FESEM; MIRA III, TESCAN) and transmission electron microscopy (TEM; Zeiss, LEO 912AB (120 kV), Germany). In 77 K, the Autosorb‐1 Quantachrome Sorptometer (USA) was used to analyze Brunauer‐Emmett‐Teller porosity and surface area. A 400 MHz spectrometer was used to capture ^1^HNMR spectra at ambient temperature in DMSO‐d_6_. ICP‐AES (inductively coupled plasma atomic emission spectroscopy) was performed on the Varian VISTA‐PRO instrument. Thermogravimetric analyses (TGA) were performed on a thermogravimetric/differential thermal analyzer (Netzsch‐TGA 209 F1) by heating at 10°C/min to 800°C.

## 3 RESULTS AND DISCUSSION

**SCHEME 2:**
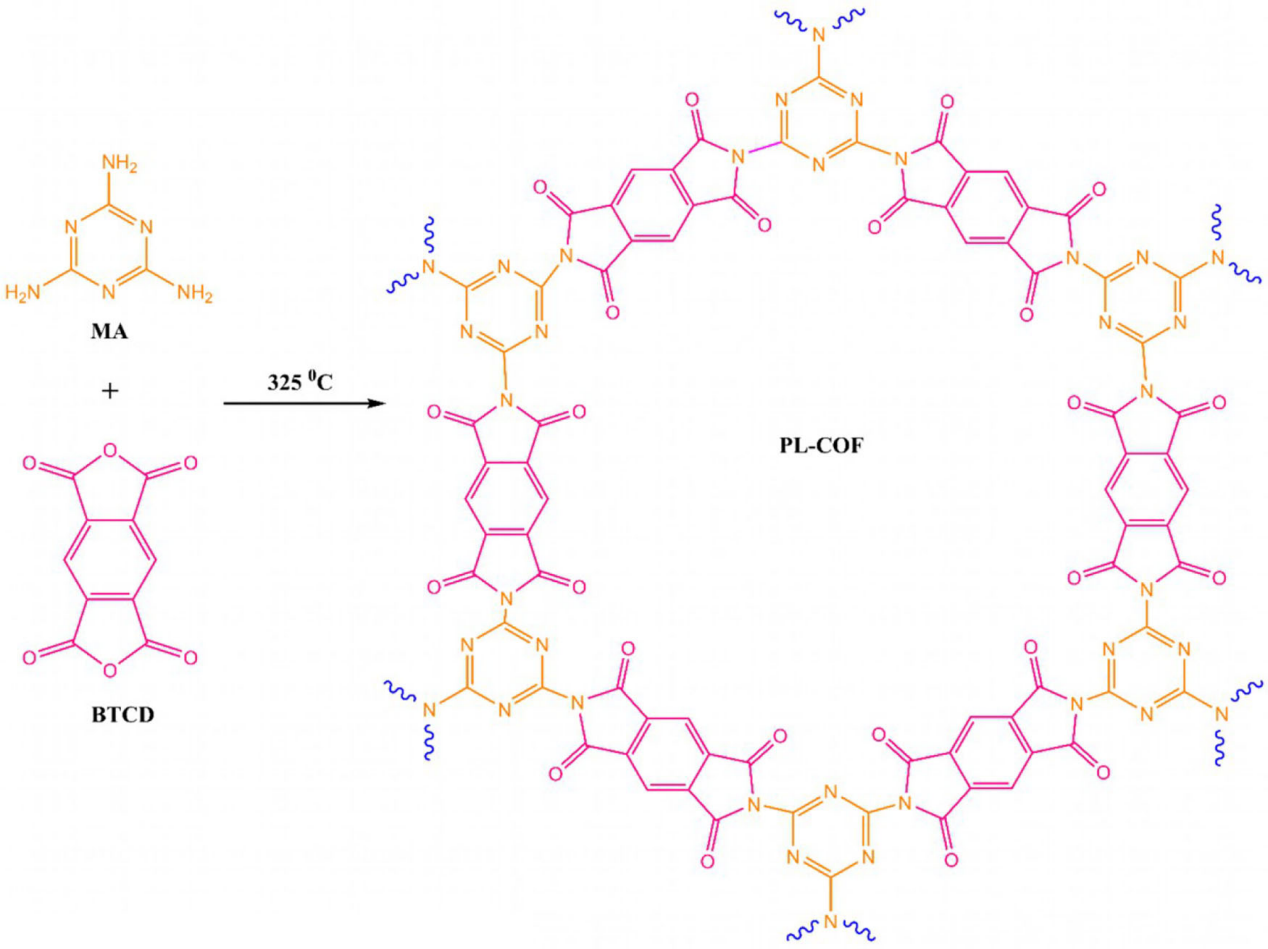
Synthesis of polyimide‐linked covalent‐organic framework (PL‐COF).

**SCHEME 3:**
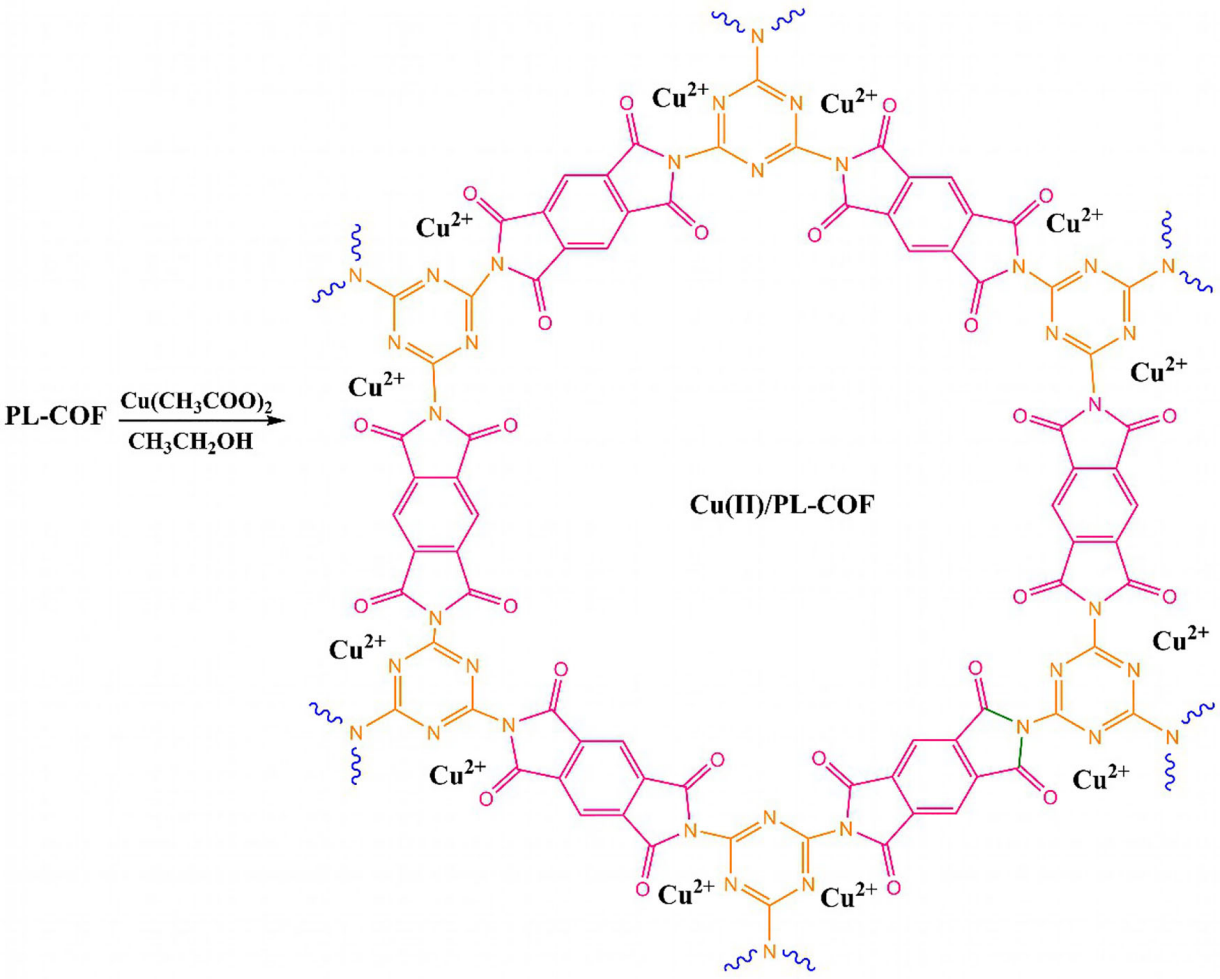
Cu(II)/polyimide‐linked covalent‐organic framework (PL‐COF) catalysis platform illustration.

The preparation route of Cu(II)/PL‐COF is shown in Schemes 2 and 3. In the experiments, melamine (MEL) and benzene‐BTCD were mixed and ground in an equimolar ratio. The mixture was then transferred to an aluminium pan and calcined at 325°C for 4 h. After air‐cooling to ambient temperature, the resulting product was rinsed with water to eliminate unreacted initial materials and finally desiccated to obtain a high yield of PL‐COF (70%). This synthetic method is relatively simple, inexpensive, and environmentally friendly as it contains no solvents and generates no dangerous waste. Owing to the porous structure, and exposure of N and O binding sites, this COF can act as a good platform for the incorporation of copper into the skeleton. Thereupon, Cu(II)/PL‐COF can be easily prepared by immersing the PL‐COF in an ethanolic solution of Cu(CH_3_COO)_2_ at ambient temperature. The total Cu incorporation was measured to be as high as 5.24 wt% by ICP‐AES.

**FIGURE 1:**
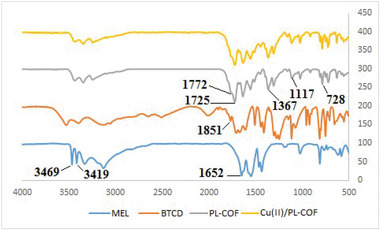
Fourier‐transform infrared (FT‐IR) spectra of melamine (MEL), benzene‐1,2:4,5‐tetracarboxylic dianhydride (BTCD), polyimide‐linked covalent‐organic framework (PL‐COF) and Cu(II)/PL‐COF.

FT‐IR spectroscopy, X‐ray diffraction (XRD), FESEM, TEM, and TGA were used to characterize Cu(II)/PL‐COF catalyst. The BET method was utilized to determine the effective surface area and the pore size distribution. Figure 1 displays the FT‐IR spectra of MEL, BTCD, PL‐COF and Cu(II)/PL‐COF. The FT‐IR spectra of the PL‐COF showed the band fade away at 1851 cm^−1^ related to the initial anhydride monomers (BTCD) (C = O symmetric stretching) and the decrease in intensity of the bands at 1652, 3419 and 3469 cm^−1^ due to the amino group terminus of the initial MEL monomer.^38^ 4 bands at 1772, 1725 (two imide carbonyl groups stretching), 1367 (C–N–C imide axial tension), 1117 (C–N–C imide transverse stretching) and 728 cm^−1^ (imide C – N – C out of plane bending) related to the specific absorption of the polyimides were detected, demonstrating that the product was fully converted to imide to form a polyimide.^39^, ^40^ However, FT‐IR spectra of Cu(II)/PL‐COF showed no visible changes in PL‐COF, indicating that Cu(CH_3_COO)_2_ does not alter the structure.

**FIGURE 2:**
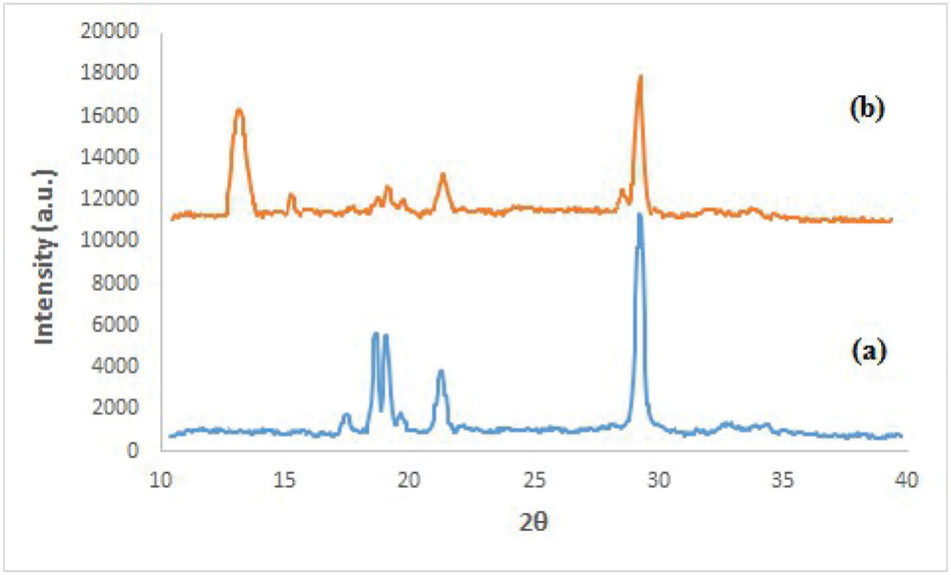
X‐ray diffraction (XRD) patterns of (A) polyimide‐linked covalent‐organic framework (PL‐COF) and (B) Cu(II)/PL‐COF.

As a result of XRD analysis of PL‐COF, diffraction peaks at 18.9 and 29.6° were observed, denoting acceptable crystallinity of the materials (Figure 2). Compared to PL‐COF, the well‐preserved XRD pattern of Cu(II)/PL‐COF has further peaks at 12.8° (2θ) and 15.2° (2θ), indicating successful loading of Cu with a minimal loss of COF integrity and crystallinity.^41^

**FIGURE 3:**
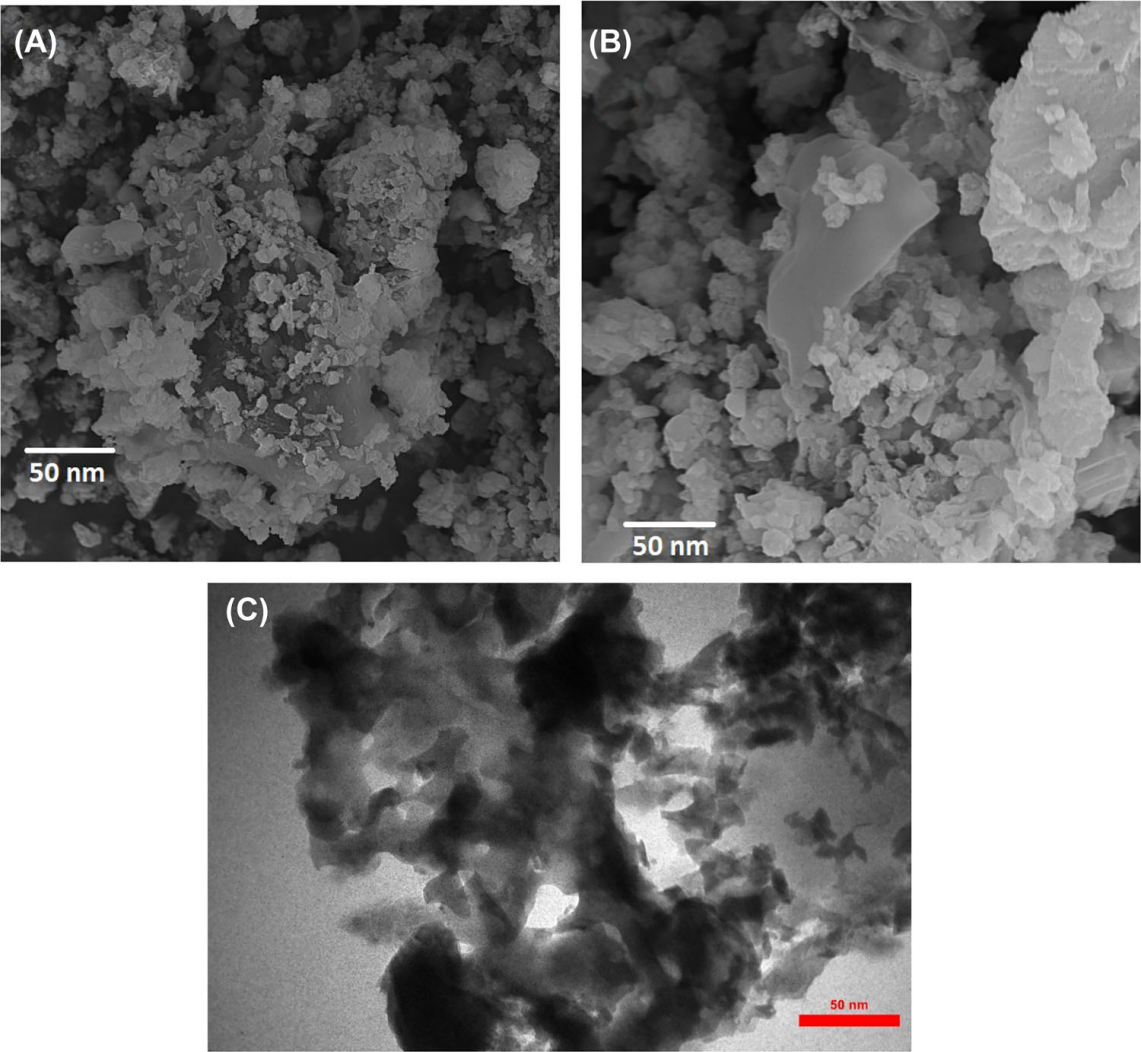
Field‐emission scanning electron microscopy (FESEM) images of (A) polyimide‐linked covalent‐organic framework (PL‐COF), (B) Cu(II)/PL‐COF and (C) transmission electron microscopy (TEM) image of PL‐COF.

The morphology of PL‐COF and Cu(II)/PL‐COF was investigated by FESEM and TEM (Figure 3). The PL‐COF and Cu(II)/PL‐COF exhibited good crystallinity and an ordered structure. The images reveal that PL‐COF possesses regular morphology, and the fundamental structure of organic frameworks seems to be the same after metal loading.

**FIGURE 4:**
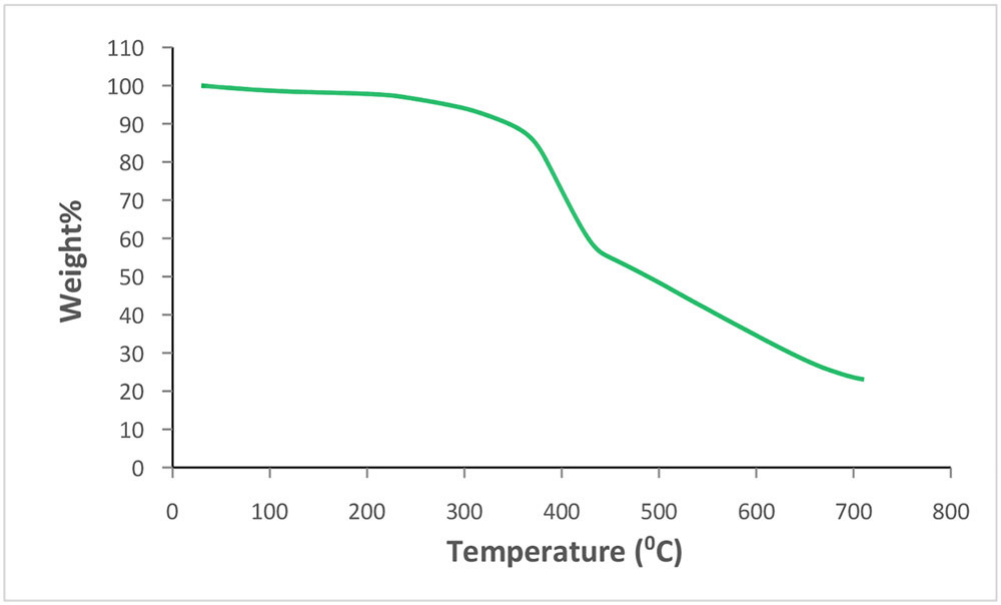
Thermogravimetric analyses (TGA) plot of Cu(II)/polyimide‐linked covalent‐organic framework (PL‐COF).

The stabilization of the catalyst is a significant factor for its practicable applications. TGA showed that Cu(II)/PL‐COF was intact up to 380°C, implying good thermostability (Figure 4).

Figure S1 illustrates N_2_ adsorption and desorption isotherms at 77 K for the PL‐COF and Cu(II)/PL‐COF composites. Pure PL‐COFs have an SSA of 43.90 m^2^/g and pore volumes of 0.035 cm^3^/g. Adsorption quantities of nitrogen gradually decrease after loading the active ingredient. Cu(II)/PL‐COF has SSA and pore volumes of 30.73 m^2^/g and 0.029 cm^3^/g, respectively. Moreover, the pore sizes measured for PL‐COF and Cu(II)/PL‐COF were 3.7 and 3.3 nm, respectively (Figure S2). Therefore, based on the above, a pore‐blocking effect may occur because Cu(II) occupies the PL‐COF pores.^42^, ^43^

After the characterization of Cu(II)/PL‐COF, its catalytic function in the multi‐component preparation of 2,4,5‐triarylimidazoles was evaluated. Optimizing the reaction conditions was the next step in the synthesis of 2,4,5‐triarylimidazoles. Therefore, the catalytic efficiency was examined during the 2,4,5‐triarylimidazoles synthesis as a model under different reaction conditions (microwave strength, catalyst dosage and time). To achieve this goal, a reaction with three components in one pot consisting of benzil **1** (1 mmol), ammonium acetate **2** (2 mmol), and aromatic aldehydes **3a‐l** (1 mmol) was chosen as the sample under solvent‐free conditions (Scheme 1). The findings were tabulated in Table 1. The findings indicated that without the Cu(II)/PL‐COF the reaction did not go forward even after 20 min (Table 1, entry 1). The results showed that the optimal conditions were reached when the reaction was accomplished in the presence of 0.05 g Cu(II)/PL‐COF under solvent‐free conditions and under microwave irradiation leading to 2,4,5‐triarylimidazole **4a** in 2 min and 96% yield (Table 1, entry 5). Remarkably, the yield of the reaction did not show a significant change with respect to conversion by increasing the Cu(II)/PL‐COF dosage up to 0.3 g (Table 1, entries 6‐8). If we reduce the Cu(II)/PL‐COF dose to 0.005 g, the yield also decreases (Table 1, entry 9). To show the performance of Cu(II) during the reaction, the performance of PL‐COF in the reaction under optimal conditions was also studied. The findings showed that when PL‐COF was used, no progression in the sample reaction was observed due to the absence of Cu(II), affirming that its presence is required for the catalysis of the reaction (Table 1, entry 13). Additionally, the sample reaction was performed with Cu(CH_3_COO)_2_ for 2 min under optimal conditions, yielding 2,4,5‐triarylimidazole **4a** with 37% yield (Table 1, entry 14). The results demonstrate that Cu(CH_3_COO)_2_ exhibits negligible performance compared to Cu(II)/PL‐COF under optimized conditions. Solvents are not considered here due to the green chemistry design.

**TABLE 1:** Evaluation of the reaction conditions for the preparation of **4a** ^a^

Entry	Catalyst (g)	Power (W)	Time (min)	Yield (%)	TON^b^	TOF^c^
1	‐	180	20	‐	‐	‐
2	Cu(II)/COF (0.05)	180	5	98	23.94	4.97
3	Cu(II)/COF (0.05)	300	5	87	21.25	4.25
4	Cu(II)/COF (0.05)	100	5	82	20.03	4.00
5	**Cu(II)/COF (0.05)**	**180**	**2**	**96**	**23.45**	**11.72**
6	Cu(II)/COF (0.1)	180	2	97	11.85	5.92
7	Cu(II)/COF (0.2)	180	2	97	5.92	2.96
8	Cu(II)/COF (0.3)	180	2	97	3.95	1.97
9	Cu(II)/COF (0.005)	180	2	30	73.28	36.64
10	Cu(II)/COF (0.01)	180	2	40	48.85	24.43
11	Cu(II)/COF (0.02)	180	2	58	35.42	17.71
12	Cu(II)/COF (0.03)	180	2	75	30.53	15.27
13	COF (0.05 g)	180	2	‐	‐	‐
14	Cu(OAc)_2_ (0.05 g)	180	2	37	0.47	0.24

^a^ Reaction condition: benzil (1 mmol), ammonium acetate (2 mmol), benzaldehyde (1 mmol) and Cu(II)/PL‐COF as a catalyst in solvent‐free condition under microwave irradiation. In terms of yields, isolated products are considered.

^b^ Turnover number

^c^ Turnover frequency = TON/time

After identifying the most suitable reaction conditions, we needed to assess the scope and efficacy of the reaction. In this respect, benzil, ammonium acetate, and aromatic aldehydes were selected to carry out the reaction to give the related 2,4,5‐triarylimidazoles derivatives (**4a‐l**) and the outcomes are depicted in Table 2. Regarding the aromatic aldehydes comprising both electron‐withdrawing and electron‐donating substituents, they can be effectively transformed to 2,4,5‐triarylimidazoles (**4a‐l**) in high yields, as indicated in Table 2. As can be seen from the data in Table 2, this procedure can be carried out for all aromatic aldehydes. It can be stated that the reaction time was significantly shortened by microwave irradiation and the products were synthesized with the best efficiency, without producing by‐products and requiring purification by column or flash chromatography. By comparing melting points and ^1^H NMR spectra with authentic samples, the products were identified.

**TABLE 2:** 2,4,5‐Triarylimidazoles **4a‐l** synthesis in the presence of Cu(II)/polyimide‐linked covalent‐organic framework (PL‐COF)

Entry	Ar	Product	Yield (%)^a^	Mp (°C)	
				Observed	Literature
1	C_6_H_5_	**4a**	96	274‐275	274‐275^23^
2	4‐Cl‐C_6_H_4_	**4b**	98	260‐261	262‐264^23^
3	4‐CH_3_‐C_6_H_4_	**4c**	96	230‐233	233‐235^44^
4	3‐NO_2_‐C_6_H_4_	**4d**	97	315‐317	317‐319^23^
5	4‐OCH_3_‐C_6_H_4_	**4e**	98	229‐230	230‐231^45^
6	2‐OH‐C_6_H_4_	**4f**	96	202‐204	202‐205^44^
7	3‐OH‐C_6_H_4_	**4g**	93	260‐261	260‐261^45^
8	4‐OH‐C_6_H_4_	**4h**	96	266‐268	266^23^
9	2‐Cl‐C_6_H_4_	**4i**	93	145‐146	190‐191^44^
10	2,4‐Dichloro‐C_6_H_3_	**4j**	95	230‐232	172‐173^23^
11	3‐Br‐C_6_H_4_	**4k**	96	302‐304	302‐304^29^
12	4‐N(CH_3_)_2_‐C_6_H_4_	**4l**	98	258‐259	256‐259^44^

^a^ In terms of yields, isolated products are considered.

**SCHEME 4:**
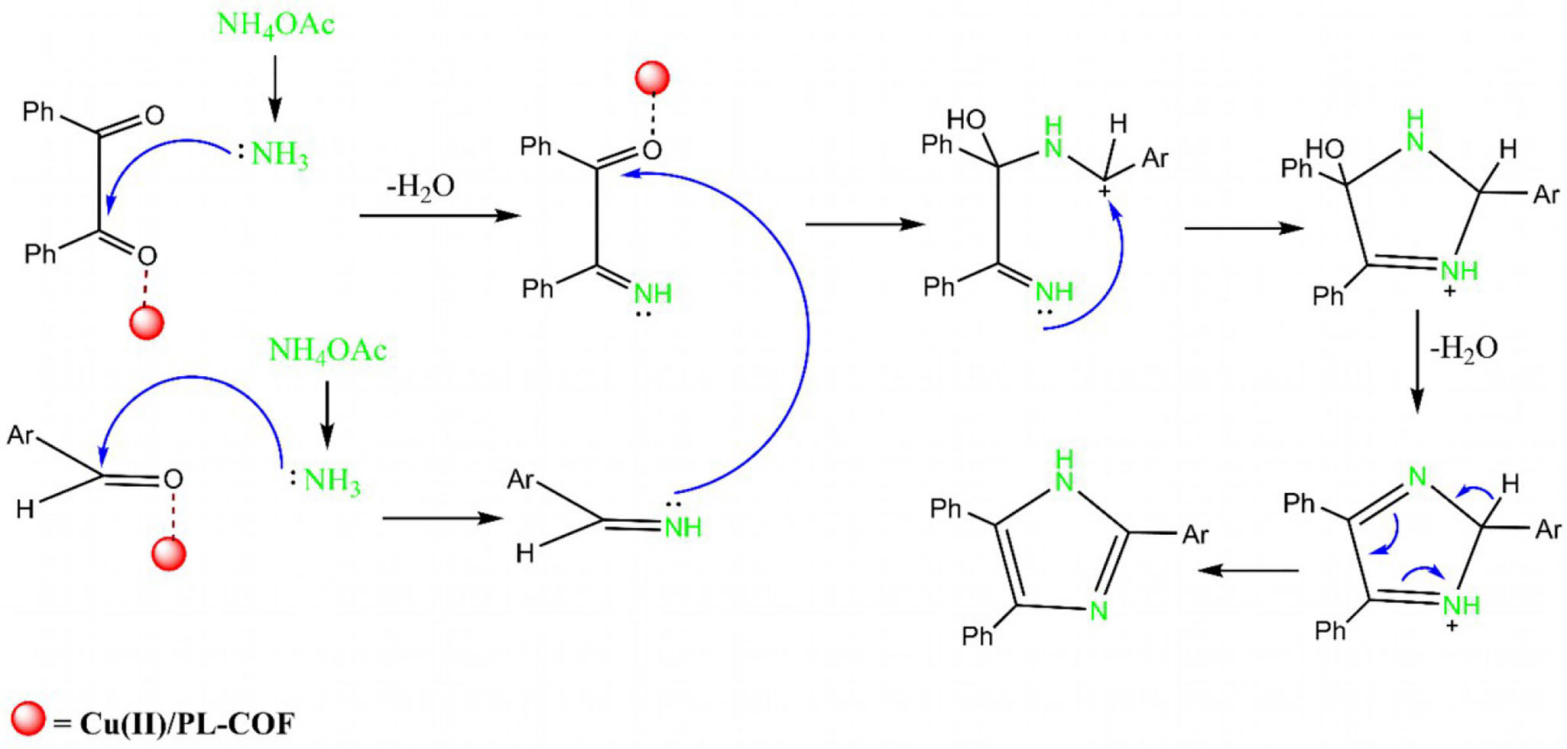
Plausible mechanism for Cu(II)/polyimide‐linked covalent‐organic framework (PL‐COF) catalyzed synthesis of 2,4,5‐triarylimidazoles.

In Scheme 4, the Cu(II)/PL‐COF catalytic activity is shown to be based on a mechanism that can be postulated. Aldehydes, as well as benzil, may be polarized by the Cu(II) ion. Nucleophilic attack of nitrogen of NH_3_ generated from NH_4_OAc on activated carbonyl produces α‐imino keone and aryl aldimine. In addition, intramolecular interaction leads to cyclization because of their subsequent reaction. To give excellent yields of trisubstituted imidazoles **4a‐l**, the formed intermediates are dehydrated to give the final product.^46^

To find whether leaching of Cu(II) occurred during the reaction, a hot filtration test was planned. This experiment was accomplished to assess the contribution of the leached active species to the reaction medium. Therefore, to a mixture of benzil (1 mmol), ammonium acetate (2 mmol,), and benzaldehyde (1 mmol), 0.05 g of Cu(II)/PL‐COF was added and the reaction was started on microwave radiation for 1 min. At this stage, the yield of the product was 50%. At that point, hot ethanol was added, and the catalyst was separated off and with the filtrate, the reaction proceeded for another 1 min. Yield% was enhanced to 55%; consequently, no significant increase in yield% was observed. This observation clearly affirmed the satisfactory steadiness of Cu(II)/PL‐COF in the reaction and no significant degradation of the catalyst occurred during the course of the reaction. ICP‐AES analysis of the filtrate indicated that the extent of Cu(II) leached into the reaction media is very small (0.4 ppm).

**FIGURE 5:**
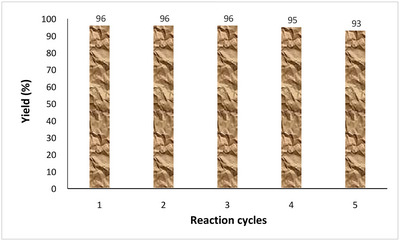
Recyclability of Cu(II)/polyimide‐linked covalent‐organic framework (PL‐COF) in the sample reaction.

The stability and recyclability of the Cu(II)/PL‐COF were confirmed in the sample reaction for the production of trisubstituted imidazole **4a**. After the reaction was complete, ethanol was added and the Cu(II)/PL‐COF was isolated by filtration, washed with acetone, and then desiccated and reused for the next experiment without appreciable loss of its catalytic performance. As shown in Figure 5, the catalyst can still be used after five cycles of continuous power.

**FIGURE 6:**
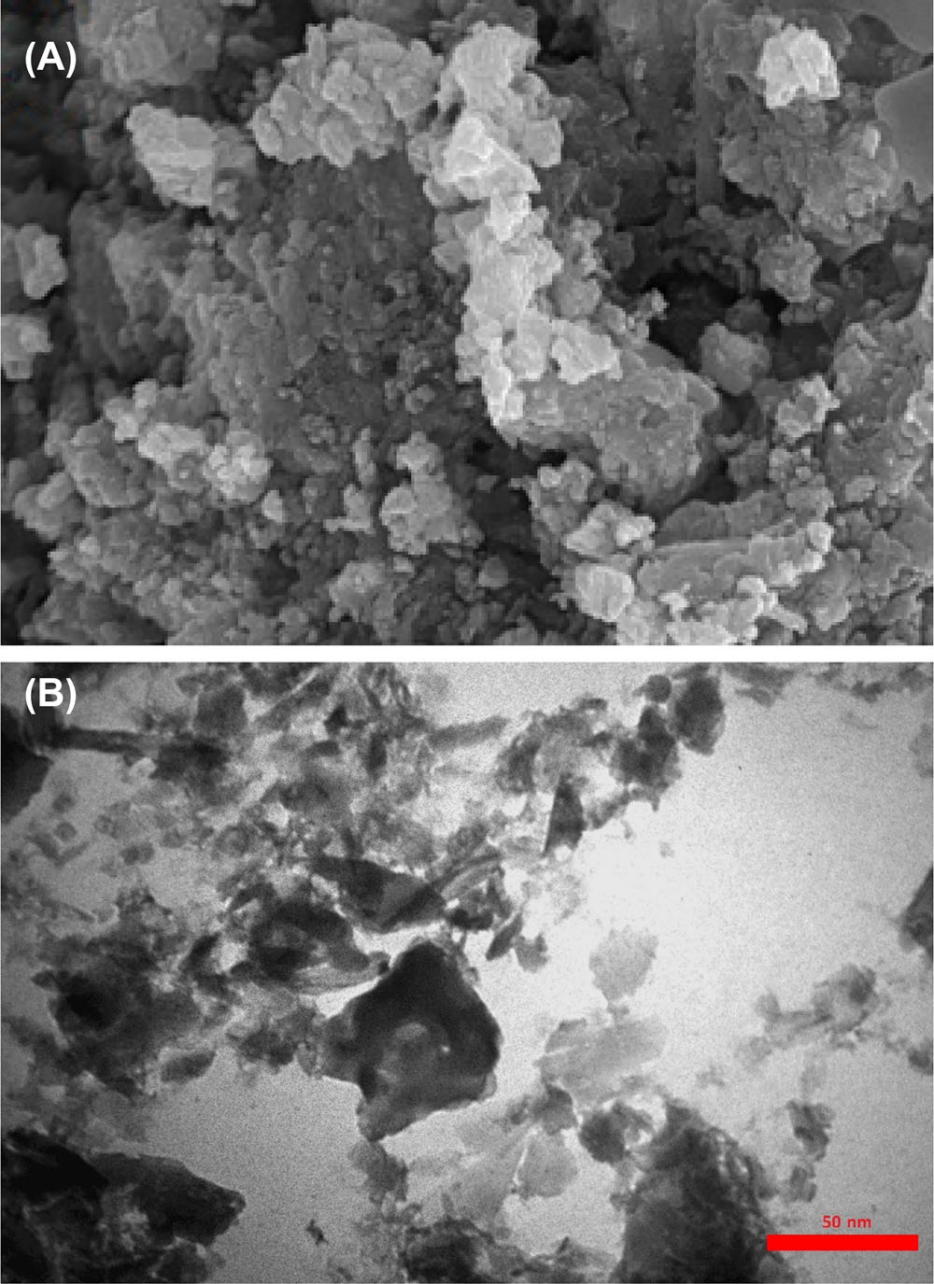
Scanning electron microscopy (SEM) (A) and transmission electron microscopy (TEM) (B) images of recycled Cu(II)/polyimide‐linked covalent‐organic framework (PL‐COF) catalyst.

The TEM and SEM pictures of the fresh and reused catalysts showed that only minor morphological changes happened (Figure 6A,B).

Table 3 compares the efficiency of the present method for the production of 2,4,5‐triarylimidazole derivatives is compared with other published papers. As illustrated in Table 3, the Cu(II)/PL‐COF catalyst has the best efficiency in the shortest time compared to the reported processes in the presence of different catalysts.

**TABLE 3:** A comparison of Cu(II)/polyimide‐linked covalent‐organic framework (PL‐COF) and other catalysts for 2,4,5‐triarylimidazole **4a** synthesis

Entry	Catalyst	Time (min)	Yield (%)^a^	Ref.
1	(NH_4_)_6_Mo_7_O_24_⋅4H_2_O	10	94	^47^ ^]^
2	MoO_3_/SiO_2_	132	95	^48^
3	Lactic acid	180	92	^49^
4	Tetrabutylammonium bromide	20	95	^46^
5	TiCl_4_‐SiO_2_	60	93	^50^
6	CoFe_2_O_4_	20	95	^29^
7	Cu(II)/PL‐COF	2	96	This study

## 4 CONCLUSION

Briefly, the use of a Cu(II)/PL‐COF catalyst under microwave irradiation facilitates the preparation of 2,4,5‐triarylimidazoles through condensation between benzil, ammonium acetate, and various aromatic aldehydes. It should be noted that this method is very simple and effective and is used to synthesize derivatives of this group of compounds. This new and effective one‐pot three‐component procedure not only features the use of microwaves and a substantial product yield, but also offers mild reaction conditions, high purity, shorter reaction times, ease of operation, reutilization of heterogeneous nanocatalysts, easy workup, and high atom economy. The association of microwave irradiation and heterogeneous catalysis has enabled the future improvement of effective, rapid, and eco‐friendly synthetic methods. The reutilization of Cu(II)/PL‐COF was high and it could be reutilized 5 times without significantly decreasing its first activity. We expect that this synthetic manner will provide better scope for the preparation of 2,4,5‐triarylimidazole analogues and will be a more viable replacement for the other available protocols.

## CONFLICT OF INTEREST STATEMENT

The authors declare no conflict of interest.

## Supporting information

Appendix A. Supporting Information
This article has been supplemented with additional information in the online version (Figures S1 and S2).

## Data Availability

Data are available on request from the authors.
