# Supplementary material for: Copper(II)/polyimide linked covalent organic framework as a powerful catalyst for the solvent‐free microwave irradiation‐based synthesis of 2,4,5‐trisubstituted imidazoles
Source: Anal Sci Adv. 2023 Apr 17;4(9-10):302–11. doi: 10.1002/ansa.202300012 (PMC10989575; doi:10.1002/ansa.202300012)
Supplement: Supplementary file 1 — Appendix A. Supporting Information This article has been supplemented with additional information in the online version (Figures S1 and S2). [file ANSA-4-302-s001.docx]

**Cu(II)/COF as a powerful catalyst for the solvent-free microwave irradiation-based synthesis of 2,4,5-trisubstituted imidazoles**

**Mahvaz Sedaghat, Farid Moeinpour *, Fatemeh S. Mohseni-Shahri**

**Supplementary information**


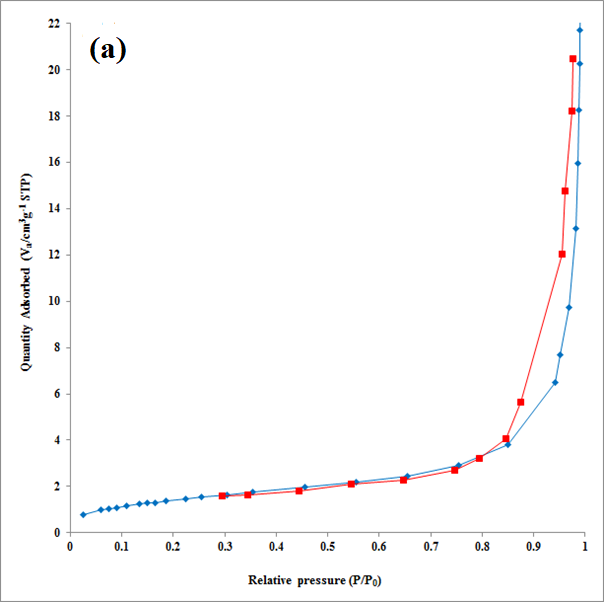


**
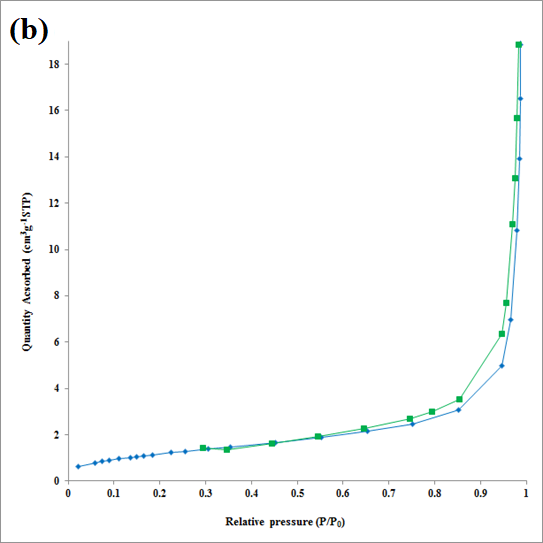
**

**Figure S1.** N_2_ adsorption−desorption isotherms of PL-COF (a) and Cu(II)/PL-COF (b)


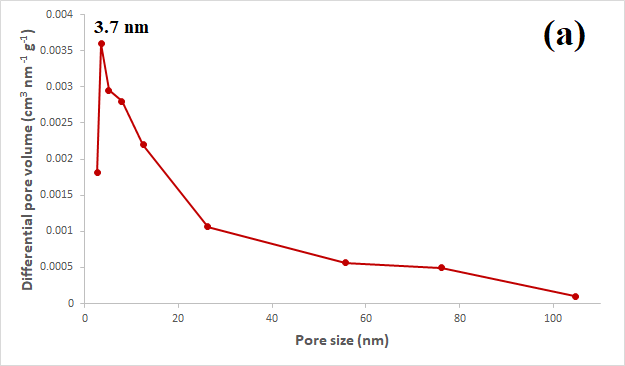


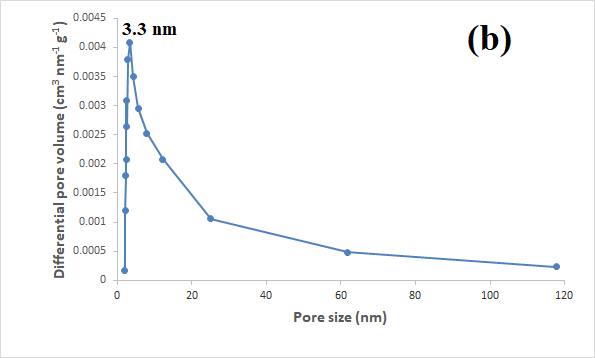


**Figure. S2** Pore size distribution of PL-COF (a) and Cu(II)/PL-COF (b)
